# Antioxidant and Antibacterial Activity, and the Amino Acid Profile of Pistachio (*Pistacia vera* L.) Waste Peptides Produced by Enzymatic Hydrolysis and Solid-State Fermentation

**DOI:** 10.3390/foods15020392

**Published:** 2026-01-22

**Authors:** Sultan Can, Hüseyin Bozkurt, Çiğdem Aykaç

**Affiliations:** Department of Food Engineering, Faculty of Engineering, University of Gaziantep, Gaziantep 27310, Türkiye; sultan@gantep.edu.tr (S.C.); aykac@gantep.edu.tr (Ç.A.)

**Keywords:** pistachio, antioxidant, amino acid, enzymatic hydrolysis, bioactive peptide

## Abstract

The pistachio nut (*Pistacia vera* L.) is a rich and high-quality source of protein, as is its waste. This study investigated the potentials of pistachio nut waste proteins to obtain bioactive peptides exhibiting antioxidative and antibacterial activities, and their amino acid profile. Enzymatic hydrolysis with pepsin, trypsin, chymotrypsin, and savinase was applied to the pistachio protein isolate (PPI) obtained from pistachio waste. In addition, solid-state fermentation (SSF) was applied to defatted pistachio with *Bacillus subtilis*, and peptides were produced. The highest degree of hydrolysis was obtained at 28.2% by using pepsin (*p* < 0.05). The highest ABTS radical scavenging activity was found as 232 µmol TE/g defatted pistachio (d.b.) for trypsin hydrolysate (*p* < 0.05). The maximum DPPH radical scavenging activity was found as 70.2 µmol TE/g defatted pistachio (d.b.) by hydrolysis with savinase. After gel filtration, the highest ABTS radical scavenging activity was found to be 0.1166 mg TE/mL in the T7 sample (*p* < 0.05), while the highest DPPH scavenging activity was found to be 0.0573 mg TE/mL in the S8 sample (*p* < 0.05). The sample showing the highest antibacterial activity was chymotrypsin hydrolysate with MIC = 0.378 mg/mL against *Staphylococcus aureus*. The total amino acid contents (TAA) of PPI, hydrolysate samples, and the SSF sample ranged from 63.136 to 76.665 g/100 g protein. It was also seen that proteins and peptides obtained from pistachio waste have a rich amino acid profile, especially Asp and Tyr, and good antioxidant activity.

## 1. Introduction

The proteins found in food not only serve as nutrients but also perform physiochemical functions that support health. The majority of the physiological activities of proteins are carried out by peptide sequences encoded in the main protein and activated by breaking the bonds in the parent protein at specific locations [1]. Bioactive peptides are described as particular protein fragments that show positive effects on the functions of the body and on health [2]. Bioactive peptides can provide health-promoting effects by interacting with enzymes, receptors, and some biomolecules in the organism due to their specificity, high tissue affinity, and effectiveness [3]. Therefore, studies of food-derived bioactive peptides are increasing exponentially. Bioactive peptides are obtained by hydrolysis occurring during the proteolysis of proteins (in vitro hydrolysis using proteolytic enzymes and gastrointestinal digestion), during food processing (cooking, ripening, and fermentation), as well as with the help of proteolytic microorganisms [1,2]. The health benefits of bioactive peptides include antimicrobial, antioxidant, antihypertensive, immunomodulatory, anticarcinogenic, enzyme inhibitory, and mineral binding effects. Bioactivity is dependent on the amino acid composition and the sequence of the peptides. The length of bioactive peptide sequences varies between 2 and 20 amino acids, and many peptides show multifunctional properties [1,4].

Bioactive peptides can be obtained from many plant and animal sources. Among nuts, pistachios are antihypertensive and antioxidant [5,6,7]; hazelnuts are antioxidant, antihypertensive, and cytoprotective [8,9]; peanuts are antioxidant and antihypertensive [10,11]; pine nuts are antioxidant and antihypertensive [12,13]; and walnuts are sources of antioxidant peptides [14,15]. Considering all these studies, it can be seen that food-derived peptides exhibit many positive bioactivities. The pistachio nut (*Pistacia vera* L.) is one of the most popular tree nuts and is valued globally because of its nutritional value, sensory and health characteristics, and economic significance. It is a member of the *Anacardiaceae* or cashew family. The pistachio is a rich source of protein (around 20%), fiber, an array of healthy fats, including mono- and polyunsaturated fatty acids, a range of micronutrients (potassium, phosphorus, and magnesium are the highest levels of minerals), and bioactive compounds such as phenolics and flavonoids [16]. In the literature, the protein content of raw pistachio is given as 20.16% [17] and 20.27% [18], while the protein content of defatted pistachio waste is reported as 33.20% [17]. It can be seen that pistachio itself and its waste are good sources of protein.

The activity of peptides and protein hydrolysates is dependent on the protein source, the process used to obtain protein from the source, the type of enzymes used, the hydrolysis conditions applied, and the final peptide concentration [2,19]. In addition, bioactivity is related to the size, nature, structure, composition, hydrophobicity level, and amino acid sequence of the peptides [11,14,20,21]. Different peptide production methods (enzymatic hydrolysis after protein isolation and solid-state fermentation) can provide peptide groups with different properties. In addition, because different enzymes break the protein at different points, different enzymes can reveal various bioactive peptide groups. In other words, different peptides can be obtained as a result of hydrolysis of a protein with different enzymes [21]. This results in obtaining a wide range of peptides. For these reasons, the method and conditions for obtaining peptides significantly affect the resulting peptide groups and therefore the bioactivities of these peptides. Moreover, since the most commonly used methods for obtaining bioactive peptides are the hydrolysis of protein molecules with proteolytic enzymes and microbial fermentation, we preferred to use enzymatic hydrolysis and solid-state fermentation methods in our study [1]. To the best of our knowledge, no study has been found in the literature investigating the bioactivities of peptides obtained from pistachio waste using different methods. It is predicted that a study in this direction will contribute to the literature, as different peptide production methods will enable peptide groups that show bioactivity in various ways to be obtained.

During the pistachio processing periods, a large amount of waste, as a powder, is produced. While pistachios to be added to desserts, candies, and chocolates are cut into small pieces, pistachio waste accumulates at the bottom of the processing line. In this study, it was investigated whether bioactive peptides could be obtained by enzymatic hydrolysis and solid-state fermentation from pistachio waste, and which peptide production method was more effective with regard to the antioxidant and antimicrobial activity of peptides.

## 2. Materials and Methods

### 2.1. Materials

Pistachio nut waste was obtained from Altın Fıstık (Gaziantep, Türkiye). Pepsin (P7000), trypsin (93615), chymotrypsin (C4129), savinase (P3111), ABTS radical (2,2′-Azino-bis(3-ethylbenzothiazoline-6-sulfonic acid) diammonium salt), DPPH radical (2,2-Diphenyl-1 picrylhydrazyl), Bradford reagent, Phthaldialdehyde reagent, Trolox (6-Hydroxy-2,5,7,8-tetramethylchroman-2-carboxylic acid), bovine serum albumin (BSA), Tris (hydroxymethyl) amino methane, and FMOC were purchased from Sigma-Aldrich (Darmstadt, Germany). The Mueller–Hinton agar and broth, 30 µg tetracycline antibiotic disks, 0.5 McFarland turbidity standard, and glass wool were purchased from Thermo Fisher Scientific (Waltham, MA, USA). Sephadex G-25 fine size was purchased from Cytiva (Wilmington, NC, USA).

### 2.2. Defatting of Pistachio Waste

Pistachio waste was ground with a Waring blender (8011ES Model HGB2WTS3, Waring Commercial, Torrington, CT, USA) to obtain a powder form. A mixture of pistachio waste and n-hexane at a ratio of 1:5 (*m*/*v*) was stirred for 3 h on magnetic stirrer at room temperature and filtered. For the second extraction, pistachio waste and n-hexane 1/5 (*m*/*v*) were mixed for two more hours on the magnetic stirrer and filtered. These filtrates were combined and hexane was removed from the pistachio waste in vacuum oven at 40 °C for 3 h to obtain defatted pistachio. It was stored at −80 °C until its usage. The protein content of defatted pistachio was determined by the Kjeldahl method and found to be 34.1%.

### 2.3. Protein Isolation

Protein isolates were obtained by alkaline extraction followed by isoelectric precipitation according to the method reported by Ferreira et al. [22] with some modifications. Ultrasound was applied to defatted pistachio mixed with distilled water in the ratio of 1:10 (*m*/*v*) at 15 Hz for 40 min to increase the extraction efficiency. After that, the pH of the mixture was adjusted to 10 with 1 M sodium hydroxide and stirred for 1 h at 600 rpm on a magnetic stirrer at room temperature. Centrifugation was applied to the mixture at 10,733× *g* at 4 °C for 20 min (Eppendorf 5810 R, Hamburg, Germany). Soluble proteins were precipitated by collecting the supernatant and adjusting the pH to 4 with 1 M HCl. The suspension was centrifuged again at 10,733× *g* at 4 °C for 20 min. Freeze-drying was applied to sediments (Christ Alpha 1-4 LD plus, Osterode, Germany) and pistachio protein isolate (PPI) was produced. The protein content of the PPI was found to be 57.80% by the Kjeldahl method.

### 2.4. Hydrolysis of Pistachio Protein Isolate (PPI)

Preliminary studies were carried out with different hydrolysis durations, E/S ratios, and initial protein concentrations for enzymatic hydrolysis, and the conditions in which the best results were obtained (the highest degree of hydrolysis and the highest protein concentration) were studied. Hydrolysis durations of 3, 6, 8, 12, 15, 18, and 24 h; E/S ratios of 1/20, 1/50, 1/100, and 1/300; and initial protein concentrations of 2, 5, 10, and 20 mg/mL were studied and the optimum conditions were found to be 1/300 E/S ratio, 6 h hydrolysis time, and 2 mg/mL initial protein concentration. The freeze-dried PPI was dissolved and then divided into four parts. Each part was hydrolyzed by use of 1/300 enzyme to substrate, E/S (*m*/*m*), ratio in a shaking incubator (Innova40, New Brunswick Eppendorf, Hamburg, Germany) at 150 rpm for 6 h at 37 °C for trypsin, pepsin, and chymotrypsin, and at 50 °C for savinase. For pepsin hydrolysis, a protein solution was prepared with 0.02 M citrate buffer (pH:2), and 0.02 M Tris-HCl buffer (pH:8) was used for the other three enzyme treatments. The hydrolysis reaction was terminated by heating in boiling water bath for 15 min. The solutions were then cooled to the room temperature in a cold water bath and centrifuged at 6037× *g* for 15 min. After that, the hydrolysis degree was determined by the o-Phthaldialdehyde reagent (OPA) method [5,6].

### 2.5. Determination of Degree of Hydrolysis

The extent of the proteolysis of the samples was measured using the OPA method. The ready-to-use OPA solution was used for the analysis. To assay the proteolysis, 400 µL of each hydrolysate was mixed with 3.0 mL of OPA solution. The solution was vortexed and incubated for exactly 2 min at room temperature. The absorbance was recorded at 340 nm using a spectrophotometer (Pharmacia Novaspec II, Uppsala, Sweden). The hydrolysis degree was calculated according to the following formula:

DH (%) = (ABS × 1.934 × d)/c

where

ABS: absorbance of the sample;
d: dilution factor;
c: protein content of the sample (g/L) [23,24,25].

### 2.6. Protein Content Determination with Bradford Assay

The Bradford reagent was mixed slowly in the bottle and brought to room temperature. Then, 1.5 mL of Bradford reagent was added to 0.05 mL of sample in each tube and they were vortexed. The sample solutions were kept in the dark for 15 min at room temperature. The Absorbances were measured at 595 nm. The protein concentration of the samples was determined using the bovine serum albumin (BSA, 0.01–0.1 mg/mL) as standard [26].

### 2.7. Peptide Separation by Gel Filtration Chromatography

Sephadex G-25 was swelled in 25 mM potassium phosphate buffer (pH: 7.4) at 20 °C overnight and packed to the column (10 × 455 mm). After packing, the column was equilibrated with 25 mM KP buffer, pH 7.4 at a flow rate of 1.0 mL/min using the peristaltic pump (Shenchen LabV1 YZ1515x, Baoding, China). During the chromatography, the elution buffer (25 mM KP buffer, pH 7.4) was pumped into the column at a speed of 1.0 mL/min. Then, 2 mL of the hydrolysates was loaded to the column and about 2 mL of filtrate fractions were collected into each tube. For each hydrolysate, 80 mL (40 tubes) filtrate fractions were collected. The filtrate fractions were named in order of arrival to the tube. For example, trypsin hydrolysates are named as T1, T2, T3 …. and T40. At the end of the separation, the column was rinsed with elution buffer for regeneration. The filtrate fractions were taken to the 2 mL eppendorf tubes and stored at −18 °C until further analysis. Firstly, the protein content of the samples was determined by the Bradford method.

### 2.8. Solid-State Fermentation with Bacillus subtilis IAM 12118

About 4 mL of sterile distilled water was added to 2 g of autoclaved defatted pistachio waste. D(+)-glucose monohydrate was added with the ratio of 15.95 mg glucose/g pistachio waste. After the activation of *Bacillus subtilis* IAM 12118, microorganisms were added as 3.94 × 10^10^ cfu/g pistachio waste. Fermentation was performed in a shaking incubator (Innova40, New Brunswick Eppendorf, Hamburg, Germany) at 150 rpm and 37 °C. The fermentation time was 48 h. After fermentation, samples were mixed with distilled water in the ratio of 1:15 (*w*/*v*) in the shaking incubator at 150 rpm, at 37 °C for 3 h 25 min. The bacteria were inactivated by placing the samples into the boiling water bath for 30 min. The mixture was cooled to room temperature. It was centrifuged at 10,733× *g* for 15 min and the resultant supernatant was recentrifuged for further 10 min. The supernatant was taken and stored at −20 °C [27,28].

### 2.9. Determination of Antioxidant Activity by DPPH• Scavenging Method

The DPPH radical scavenging activity was determined according to the method proposed by Brand-Williams et al. [29]. In total, 500 μL of extract or blank were added to a 2500 μL of a 89.7 μmol/L (final absorption adjusted to 0.800 ± 0.010 AU at 517 nm) DPPH• ethanolic solution. All solutions were kept in dark and the absorbance was measured at 517 nm against a 95% ethanol blank after 1 h reaction time. Trolox solutions were prepared to draw the calibration curve. The antioxidant activity values were expressed as mg Trolox equivalents (TE) per ml of sample or µmol TE/g defatted pistachio (d.b). All measurements were made in triplicate.

### 2.10. Determination of Antioxidant Activity by ABTS Radical Scavenging Activity Method

In total, 7 mM ABTS aqueous solution and 2.45 mM K_2_S_2_O_8_ aqueous solution were mixed in equal volume. ABTS radical was formed by keeping this mixture in the dark environment at room temperature for 12–16 h. Then, 50 mM of phosphate-buffered solution (PBS, pH 7.4) was used to dilute ABTS solution to obtain the absorbance of 0.70 ± 0.03 at 734 nm. The sample and Trolox were diluted with the PBS solution. Then, 3 mL of diluted ABTS radical solution was added to 0.5 mL of sample and after 1 h reaction time, and the absorbance was determined by spectrophotometer at 734 nm. The PBS solution and Trolox were used as the control and standard antioxidant, respectively. The calibration curve was plotted with Trolox prepared at different concentrations, and, according to this curve, the Trolox equivalent antioxidant capacity (TEAC) of the samples was calculated and expressed as a milligram of Trolox equivalents per milliliter of sample (mg TE eqv/mL sample) or µmol TE/g defatted pistachio (d.b). All the tests were performed in triplicate [15,30].

### 2.11. Determination of Antibacterial Activity

The antibacterial activity of the samples against *Escherichia coli* ATCC 25922 and *Staphylococcus aureus* ATCC 25923 was determined by the agar well diffusion method and broth macro dilution method. Similarly to the procedure applied in the disk diffusion method, the Mueller–Hinton Agar (MHA) plate surface is inoculated by spreading a 100 µL of the microbial inoculum over the entire agar surface. Inoculum was prepared by making a saline suspension of isolated colonies selected from 18 h incubated nutrient agar plate. The suspension was prepared as turbidity equivalent to the 0.5 McFarland standard. This results in a suspension containing approximately 1 × 10^8^ colony-forming units (CFU)/mL for microorganisms. Then a hole with a diameter of 8 mm was punched aseptically with a sterile pipet tip and a few drops of liquid MHA were dropped at the bottom of the well. After liquid MHA solidified, 100 µL of the sample solution was added into the well. Then, MHA plates were incubated at 37 °C for 16 h. The antimicrobial agent diffuses in the agar medium and inhibits the growth of the microbial strain. The inhibition zone diameters were measured at the end of the incubation time. Sterile distilled water was used as the negative control and tetracycline as the positive control [31,32].

The broth macro dilution method was used to determine the minimum inhibitory concentration (MIC) of the samples as recommended by the Clinical and Laboratory Standards Institute [33]. About 2 mL of the sample solutions were used in two-fold dilution with tubes already containing 2 mL of Mueller–Hinton broth to obtain the final concentrations. For each sample serial dilutions of protein concentrations were studied. Then, 100 μL of the standard bacterial inoculums (corresponding to 5 × 10^4^ cfu) were added into the tubes and mixed well. The tubes were incubated at 37 °C for 24 h for bacteria. The MIC was determined as the lowest sample concentration, resulting in no visible turbidity in the tubes.

### 2.12. Determination of Amino Acid Profile

The amino acid composition was determined by high-performance liquid chromatography (HPLC, Shimadzu, Tokyo, Japan) equipped with a fluorescence detector (RF-20A) and a C18 column (250 × 4.6 mm, Tokyo, Japan). Detection was carried out using two channels (CH1: 340/450 nm, CH2: 266/305 nm), following the procedure described by Jajić et al. [34] with modifications according to Kabelová et al. [35]. Derivatization was performed automatically by the autosampler using borate buffer, OPA, FMOC, and ultrapure water. A 10 µL aliquot of the sample was injected, and the separation was conducted at 40 °C. The solvent gradient (% volume) was programmed as follows: 0 min: 0% B; 1.9 min: 0% B; 18.1 min: 57% B; 18.6 min: 100% B; 22.3 min: 100% B; 23.2 min: 0% B; and 26 min: 0% B. Standard amino acid solutions were prepared in 0.1 M HCl at different concentrations and subjected to the same derivatization and chromatographic conditions. Calibration curves were constructed for each amino acid, and quantification was expressed in milligrams per liter (ppm). Since all analytes were derivatized, they were detected at the same wavelength. All analyses were performed in triplicate.

### 2.13. Statistical Analyses

Statistical analyses were made using SPSS software version 22.0 (IBM Corporation, Armonk, NY, USA). The differences between samples were assessed using one-way ANOVA for protein content, hydrolysis degree, DPPH, and ABTS radical scavenging activities of samples. The Shapiro–Wilk test was applied for normality testing prior to applying one-way ANOVA. The Duncan multiple range test was carried out to find a statistically significant group at α = 0.05 level. For correlation analysis, Pearson’s correlation was performed. Principle component analysis (PCA) was applied using Origin Lab software version 2018 (Northampton, MA, USA).

## 3. Results and Discussion

### 3.1. Degree of Hydrolysis

The properties of the protein, the type of enzyme used, the enzyme/substrate ratio, temperature, and hydrolysis duration influence the hydrolysis degree of the protein and the type of peptide produced [1]. The hydrolysis of a protein with different peptidases results in the production of different peptide structures. Different peptides exhibit different functional and bioactive properties [5,19,21]. It was found that there was no significant (*p* > 0.05) difference between the hydrolysis degree of trypsin, chymotrypsin, and savinase hydrolysates, whereas it was the highest in pepsin treatment (*p* < 0.05) as 28.2% (Table 1). When wild pistachio protein was hydrolyzed with trypsin, about a 39.7% hydrolysis degree was obtained, and for pepsin hydrolysate the hydrolysis degree was reported as 26.9% [7]. For pepsin hydrolysis, a similar result was obtained in this study despite the different enzyme-to-substrate ratio used. For trypsin hydrolysis, the treatment time and E/S ratio were different from this study. These factors affect the degree of hydrolysis. In the enzymatic hydrolysis of wild almonds, the maximum degree of hydrolysis was obtained with alcalase while the minimum with pepsin [19]. Chickpea protein concentrate was hydrolyzed with alcalase by Ghribi et al. [36]. They found that the hydrolysis degree was 14.7% after 220 min of hydrolysis. In the hydrolysis of peanut protein isolate with alcalase, 40.0% hydrolysis degree was reached after 22 h [10]. It can be concluded that type of the nut, the enzyme, and the duration affected the hydrolysis degree.

### 3.2. Protein Content

Among all pre-gel samples, the sample with the highest protein concentration was PPI, while the sample with the lowest concentration was pepsin hydrolysate and the concentrations were found to be 31.23 mg/mL and 0.45 mg/mL, respectively (*p* < 0.05) (Table 2). Before analysis, extraction was applied to the defatted pistachio at pH:10 at 600 rpm for 1 h and the protein concentration of the extract was found to be 19.00 mg/mL. In the study where nut proteins, including pistachio, were extracted with 50 mM Tris-HCl (pH: 7.5), it was found that 30 mg nut sample contained 4–8 mg protein [37]. The difference between protein amounts could be caused by solvents used. Solubility and extractability vary in different solvents. Different solvents and buffer solutions extract different components of the protein [38]. Applied pretreatments and adjusted pH also affect the extracted amount of protein. The protein concentration of hydrolysates was found to be between 0.45 and 1.51 mg/mL, while the protein concentration of pepsin hydrolysate (0.45 mg/mL) was found to be significantly lower (*p* < 0.05) and the protein concentration of chymotrypsin hydrolysate (1.51 mg/mL) was significantly higher (*p* < 0.05). The protein contents of savinase and trypsin hydrolysates did not show any significant difference between themselves (*p* < 0.05) (Table 1). It can be concluded that type of enzyme used is effective in terms of impacting protein concentration. The protein concentrations of the samples after gel filtration were determined by the Bradford method. Depending on the bead material (Sephadex G-25 in our study) used in gel filtration, a fractionation is made based on the molecular weight of the peptides. Sephadex G-25 has a fractionation range for globular proteins of molecular weights 1000 to 5000 Da. Peptides higher than molecular weights of 5000 Da cannot enter the pores and elute earlier from the gel matrix (earlier tubes). Peptides with molecular weights lower than 5000 Da can enter the gel matrix’s internal pores, traveling a longer path related to their size and resulting in a longer elution time (medium and later tubes), are therefore easily separated from peptides with molecular weights of less than 1000 Da. The uppermost protein concentrations were detected in S8, T6, and C8 fractions as 0.3302 mg/mL, 0.3123 mg/mL, and 0.3096 mg/mL, respectively (Tables S1 and S2 in Supplementary Materials). When different protease enzymes are used to hydrolyze PPI, it can be said that different peptide distributions obtained results in the peptides being collected in different tubes showing different protein concentrations. When trypsin was used, four fractions with the highest protein concentration were obtained (T6 to T9), while three fractions (C8 to C10) were obtained when chymotrypsin was used, two fractions (P9, P10) were obtained when pepsin was used, and five fractions (S8 to S12) were obtained when savinase was used.

### 3.3. Antioxidant Activity

Protein hydrolysates are important sources of antioxidants. The antioxidant activity of the hydrolysate is related to the size (length), nature, structure, composition, hydrophobicity level, and the amino acid sequence of the peptides [11,14,20,21]. Peptide groups with different properties can be produced with different peptide production methods. Therefore, peptide production conditions significantly affect the produced peptides, and so affect the bioactivities of these peptides. The type and structure of amino acids play a significant role in impacting the antioxidant activity of the peptide [21].

The highest ABTS radical scavenging activity (*p* < 0.05) was seen to be 232 µmol TE/g defatted pistachio (corresponds to 87%, 1.0 mg TE/mL) in trypsin hydrolysate, while the highest DPPH radical scavenging activity was seen to be 70.2 µmol TE/g defatted pistachio (55.0%, 0.2 mg TE/mL) in savinase hydrolysate (*p* > 0.05) (Table 2). In the literature, where pistachio proteins were hydrolyzed with trypsin and chymotrypsin, the highest DPPH radical scavenging activity was found to be 83.7% in the chymotrypsin hydrolysate sample [5]. Sarabandi et al. [7] hydrolyzed PPI with pepsin, alcalase, pancreatin, and trypsin. The highest DPPH and ABTS radical scavenging activity was observed for alcalase hydrolysate. While DPPH radical scavenging activities were determined to be between 55.6% and 64.2%, ABTS radical scavenging activities were determined to be between 71% and 90%. The difference among antioxidant activities could be due to the fact that different enzymes can break proteins from different regions, thus allowing peptides with different structures and properties to be obtained. For this reason, peptides with different levels of antioxidant activities are obtained by hydrolysis with different enzymes. The type of used enzyme affects the type and magnitude of bioactivity shown [1]. In this study, while SSF with *Bacillus subtilis* showed 209 µmol TE/g defatted pistachio ABTS radical scavenging activity, the DPPH radical scavenging activity was 35.8 µmol TE/g defatted pistachio. Bioactive peptides obtained by the SSF of corn gluten meal with *Bacillus subtilis* MTCC5480 showed in vivo antioxidant activity [26,27,28]. Peptides obtained after the SSF of walnut protein meal by *Bacillus subtilis* showed a DPPH radical scavenging ability of 87.8% [27]. Considering other studies, it can also be said that applying SSF to food proteins is a good method to obtain natural antioxidant peptides.

In this study, ABTS and DPPH radical scavenging activities of defatted pistachio were found to be higher than the activities of PPI. It was found that the ABTS radical scavenging activity of defatted pistachio was 198 µmol TE/g of defatted pistachio (74%, 102 µmol TE/g nut), while the activity of PPI was 53 µmol TE/g of defatted pistachio (21%, 27 µmol TE/g nut). DPPH radical scavenging activity was determined as 60.6 µmol TE/g defatted pistachio (54.1%) for defatted pistachio, while it was determined as 15.7 µmol TE/g defatted pistachio (14.3%) for PPI. It can be thought that the decrease in activities after the protein was isolated was caused by the isolation process. The isolation process may have caused the loss of some antioxidant components. In the study conducted by Sarabandi et al. [7], PPI showed approximately 40.0% DPPH radical scavenging activity, while it showed approximately 30% ABTS radical scavenging activity. They used phosphate-buffered saline as the extraction solvent. The differing extraction solvents may extract proteins that show different levels of antioxidant activities. The ABTS radical scavenging activity of pistachio was found to be 36 µmol TE/g nut by D’Evoli et al. [36]. The difference between activities may be due to the characteristics of the samples used. While the waste from the pistachio kernel was used in our study, whole unshelled pistachios were used in the study by D’Evoli et al. [39]. Gentile et al. [40] determined the ABTS radical scavenging activity of pistachio powder as 28 µmol of Trolox equivalents/g nut. The value obtained in this study is higher. This may be caused by the fact that the regions where pistachio nuts grow are different (Gaziantep and Sicilia) and the waste contains more inner shell than pistachio. Derbyshire et al. [16] stated that most of the phenolics in pistachio nuts are inside the inner shell. This may increase the antioxidant effect with the increase in the inner shell ratio.

It was determined that defatted pistachio, PPI, enzyme hydrolysates, and peptides obtained by SSF showed antioxidant activity. These findings indicate that pistachio waste could be a good source of antioxidants. In addition, the fact that the hydrolysates and SSF sample show higher ABTS and DPPH radical scavenging activities than PPI shows that enzymatic hydrolysis and SSF can be efficient methods to obtain bioactive components from proteins.

Gel filtration is an efficient method in the separation of peptides by molecular size and has been widely applied in mixed component separations, removing salts and impurities, and separating enzymes, proteins, and amino acids. The antioxidant activity in hydrolysate fractions obtained by gel filtration was determined by ABTS and DPPH radical scavenging activity methods. Antioxidant activity values were expressed as mg Trolox equivalents per ml of sample. In post-gel samples, the highest ABTS radical scavenging activity was found to be 0.1166 mg TE/mL (80.15%) in the T7 sample, and the lowest was 0.0003 mg TE/mL in the T22 sample (Table S1 in Supplementary Materials), whereas the uppermost DPPH radical scavenging activity was in sample S8 at 0.0573 mg TE/mL, and the lowest was found in the T23 sample at 0.0163 mg TE/mL (*p* < 0.05) (Tables S1 and S2 in Supplementary Materials). While the highest ABTS radical scavenging activity was seen in the T7, T8, and T6 samples for trypsin hydrolysate, respectively, for chymotrypsin in samples C8, C10, and C11, and for pepsin hydrolysate in the P11, P16, and P10 samples, for savinase hydrolysate, it was seen in samples S8, S10, and S9 (Tables S1 and S2 in Supplementary Materials). The maximum DPPH activity was seen in the T7, T6, and T8 samples for trypsin hydrolysate, respectively; for chymotrypsin, it was seen in the C8, C9, and C10 samples. While this activity was determined to be the highest for pepsin hydrolysate in the P16, P15, and P17 samples, for savinase, it was seen in samples S8, S9, and S11. When the results were examined, it can be said that the greatest antioxidant activities were seen in the first tubes containing high molecular weight peptides for trypsin, chymotrypsin, and savinase hydrolysate. It can be evaluated that the highest activities in pepsin hydrolysate are seen in the middle rows of tubes, where the peptides with medium molecular weight are. Additionally, when considering a particular enzyme hydrolysate, it can be said that the maximum activities are seen in peptides with similar molecular weight (because of the fact that the highest activities were obtained in consecutive tubes). Considering the results of the study, it can be thought that these tube orders are different for different enzyme hydrolysates and the enzyme type influences the post-gel antioxidant activity.

Pecan protein isolate was hydrolyzed with alcalase by Hu et al. [41]. The purified fraction showed scavenging activities on ABTS radical as 67.67% and DPPH radical as 56.25%. Similarly, in the post-gel savinase samples, the highest ABTS radical scavenging activity was found to be 73.81% (0.0797 mg TE/mL) while the DPPH activity was 61.89% (0.0573 mg TE/mL) in the S8 sample in our study. Our antioxidant activities were found to be higher than this study. This difference could be caused by differences in samples, enzymes used, and gel materials used. Pecan protein and pistachio protein may have different antioxidant constituents. Alcalase and savinase have different hydrolysis behaviors. As a result of this, peptide groups with different antioxidant activities and powers may have been formed. In the literature, it was stated that different peptides can be obtained as a result of the hydrolysis of a protein with different enzymes [21]. In addition, Sephadex G-25 and G-50 separate peptides of different sizes. The size of the peptides may also be a factor affecting antioxidant activity. Bioactivity is related to the size, nature, structure, composition, hydrophobicity level, and amino acid sequence of the peptides [11,14,20,21]. In another study, the enzymatic hydrolysis of chickpea protein concentrate with alcalase was performed. This hydrolysate was separated by gel filtration chromatography on a Sephadex G-25 into four main fractions. Fraction 3 showed the highest DPPH scavenging activity as 54% [36]. The major reason for the difference between the DPPH activities may be the use of different samples which are PPI and chickpea protein concentrate. In the study conducted by Ji et al. [11], peanut protein isolate was hydrolyzed with alcalase. After gel filtration (Sephadex G-15) of the hydrolysate, fractions that have antioxidant capacity (reducing power) were obtained. Considering our study and the studies in the literature, it can be said that the antioxidant activity of the fractions varies depending on the protein source, enzyme used, and gel material used. In addition, it can be said that gel filtration is a good method for obtaining antioxidant peptides by separating hydrolysates into fractions.

### 3.4. Principal Component Analysis (PCA)

PCA was applied to analyze the relationships between the different variables and determine the optimum number of extracted principal components.

**Figure 1:**
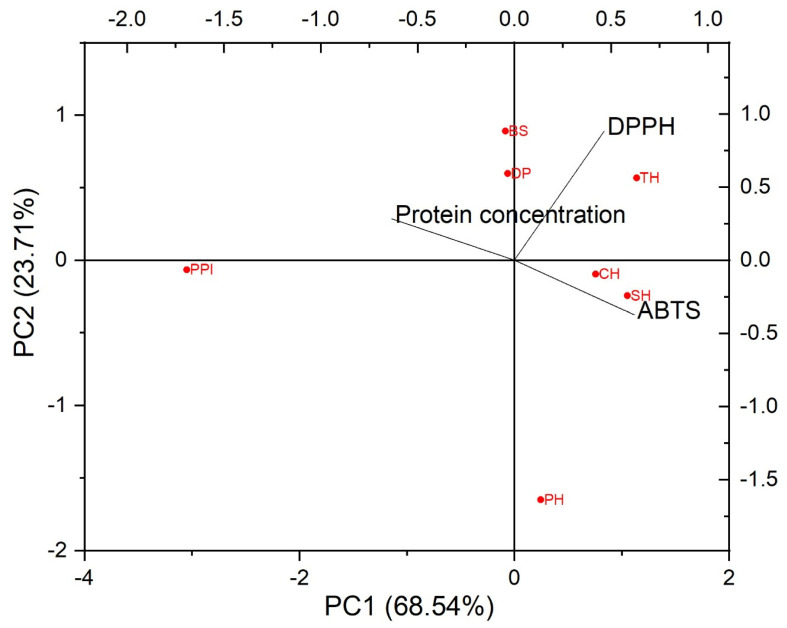
The PCA biplot indicating the correlation of the variables and the scatter plot of the observations. PC1, the first factor of principle component; PC2, the second factor of principle component. PH, pepsin hydrolysate; TH, trypsin hydrolysate; CH, chymotrypsin hydrolysate; SH, savinase hydrolysate; BS, SSF sample with *Bacillus subtilis*; DP, defatted pistachio; and PPI, pistachio protein isolate.

The first two principal components (PC1 and PC2) represented 68.54% and 23.71% of the variances of protein concentration, ABTS and DPPH radical scavenging activities, providing a good approximation of the variation present in the data. PC1 showed a high correlation with ABTS and protein concentration. Additionally, PC2 is correlated with DPPH radical scavenging activity, showing the variation between samples with regard to DPPH radical scavenging activity. DPPH radical scavenging activity has a moderate correlation with ABTS radical scavenging activity. According to the PCA biplot, hydrolysate samples (PH, TH, CH, and SH), which have high antioxidant activities, were on the right side of the PC1. The savinase hydrolysate (SH) and trypsin hydrolysate (TH) samples with the highest DPPH radical scavenging activity were located furthest to the right along PC1 (Figure 1). PPI (pistachio protein isolate), with the lowest ABTS and DPPH activities, was located at the far left of PC1.

**Figure 2:**
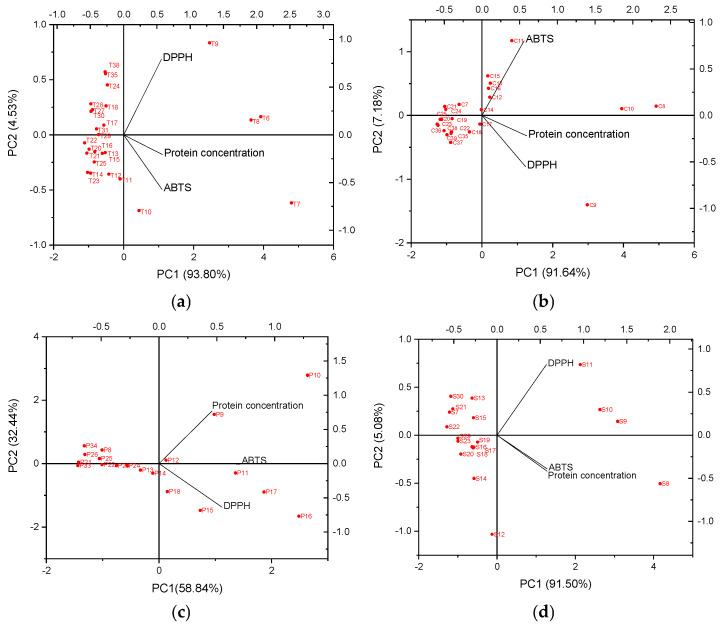
The PCA biplot indicating the correlation of the variables and the scatter plot of the observations for post-gel (**a**) trypsin hydrolysate, (**b**) chymotrypsin hydrolysate, (**c**) pepsin hydrolysate, and (**d**) savinase hydrolysate samples.

For post-gel trypsin hydrolysate samples, the first two principal components (PC1 and PC2) represented 93.80% and 4.53% of the variances of protein concentration, ABTS and DPPH radical scavenging activities, providing a good approximation of the variation present in the data. For chymotrypsin hydrolysate samples, PC1 and PC2 represented 91.64% and 7.18% of the variance. For both samples, PC1 showed a high correlation with protein concentration (Figure 2a,b). In the PCA plot of trypsin hydrolysate samples, the angle between the regression vectors of ABTS radical scavenging activity and protein concentration indicates the strong positive relationship with each other (Figure 2a). The results were in accordance with the Pearson correlation analysis. There is a positive and significant relationship between protein concentration and ABTS radical scavenging activity (r = 0.953 *p* < 0.01). Additionally, PC2 of trypsin samples is correlated with DPPH radical scavenging activity while PC2 of chymotrypsin samples is correlated with ABTS radical scavenging activity, showing the variation between samples with regard to radical scavenging activities. According to the PCA biplots, the T7 and C8 samples with the highest protein concentration and strongest DPPH and ABTS radical scavenging activities were situated furthest to the right along PC1 (Figure 2a,b). The samples with low levels of protein concentration and weak scavenging activities against DPPH and ABTS radicals were on the left side of PC1. T6, T7, T8, T9, and T10 and C8, C9, C10, and C11 located on the positive parts of the PC1 were recognized with their relatively high protein concentration, and ABTS and DPPH values (Figure 2a,b).

For pepsin hydrolysate samples PC1 and PC2 represented 58.84% and 32.44% of the variance; for savinase, PC1 was 91.50% and PC2 was 5.08% of the variance. In both samples, PC1 showed a high correlation with ABTS radical scavenging activity. In the savinase sample, PC1 also showed a correlation with the protein concentration. In the PCA plot of pepsin samples, according to the angle between the regression vectors of ABTS and DPPH radical scavenging activity and protein concentration, there is a moderate correlation between them (Figure 2c). PC2 is correlated with DPPH radical scavenging activity for both samples. According to the PCA biplot, the P10 sample with the highest protein concentration was located at the rightmost along PC1. The S8 sample with the maximum protein concentration and antioxidant activities was positioned on the right. The strong positive relationship between ABTS radical scavenging activity and protein concentration can be seen from the PCA plot of savinase (Figure 2d). This is also supported by the Pearson correlation analysis. There is a positive and significant relationship between protein concentration and ABTS radical scavenging activity (r = 0.933 *p* < 0.01). The samples P9, P10, P11, P16, and P17 and S8, S9, S10, and S11 with high protein content and antioxidant activities was located on the positive part of the PC1.

### 3.5. Amino Acid Profile Analysis

The amino acid composition of the PPI, its hydrolysates, and solid-state fermented (SSF) samples were analyzed, and the results are presented in Table 3 together with the amount of essential amino acids (EAA).

The total amino acid (TAA) contents of PPI, hydrolysate samples, and the SSF samples ranged from 63.136 to 76.665 g/100 g protein, and it was indicated that PPI was rich in Asp and Tyr. The EAA amounts of all hydrolysates and SSF samples ranged from 31.872 to 44.272 g/100 g protein, which was higher than that of PPI (29.985 g/100 g protein). Similarly, in the study conducted by Sarabandi et al. [7], the EAA amount of hydrolysates was found to be higher than pistachio protein, and protein isolate was rich in Asp. These findings are supported by a previous study by Zheng et al. [42]. In that study, peanut protein isolate hydrolyzed by flavorzyme and the EAA amount of the hydrolysate (36.97%) was found to be higher than that of peanut protein isolate (29.30%). EAA amounts were found to be similar both Sarabandi et al. [7] (28.76 g/100 g protein) and this study (29.985 g/100 g protein). The maximum amount of TAA (76.665 g/100 g protein) and EAA (44.272 g/100 g) were obtained in trypsin hydrolysate. The amino acids most abundant in trypsin hydrolysate were Tyr, Asp, and Glu, respectively. The EAA that were found in the highest amounts were Tyr, Leu, and Val. Similarly, the amino acids found in the maximum amounts in peanut protein hydrolysate were Glu, Asp, and Arg [42]. Additionally, the sample showing the highest ABTS radical scavenging activity was trypsin hydrolysate (Table 2). Peptides containing one or more amino acids, such as cysteine, methionine, tryptophan, tyrosine, phenylalanine, histidine, valine, leucine, isoleucine, and lysine, show stronger antioxidant activities. Carboxyl groups in acidic amino acids (e.g., aspartic acid and glutamic acid) may show antioxidant activity by functioning as H+ donors [19]. The antioxidant activity shown by the samples in this study can be associated with the antioxidant amino acids they contain. In addition, it can be said that proteins and peptides from nuts, rich in amino acids, are good sources of antioxidants. Considering the WHO’s recommended daily intake of EAA per kg for adults (39 mg Leu/kg per day; 26 mg Val/kg per day), the contributions of nut consumption in terms of nutrition and health are noteworthy [43].

### 3.6. Antibacterial Activity

The antibacterial activity of the samples was investigated against *Escherichia coli* ATCC 25922 and *Staphylococcus aureus* ATCC 25923 by the agar well diffusion method and broth macro dilution method. The activity results were evaluated according to whether there was microbial growth in the well and MIC values. The results of the broth macro dilution method showed that PPI was the only sample that showed an inhibitory effect against *E. coli* (MIC = 7.808 mg/mL). PPI, chymotrypsin hydrolysate, savinase hydrolysate, and the SSF sample with *B. subtilis* showed an inhibitory effect against *S. aureus.* The sample showing the highest inhibitory effect against *S. aureus* was chymotrypsin hydrolysate, with MIC value of 0.378 mg/mL. Savinase hydrolysate had an MIC value of 0.620 mg/mL against *S. aureus*, while PPI had a MIC value of 3.904 mg/mL, and the SSF sample with *B. subtilis* was found to have a MIC value of 0.776 mg/mL.

In the agar well diffusion method, the (-) symbol was used for samples showing growth in the well, and the (+) symbol was used for samples without growth (having antibacterial activity). The Results showed that defatted pistachio and all four enzymes hydrolysates showed an inhibitory effect against *E. coli.* For *S. aureus*, PPI, defatted pistachio, and all enzymes hydrolysates were effective (Table 4). In the study performed by Dumandan et al. [5], in which the extracted pistachio proteins were hydrolyzed with trypsin and chymotrypsin, the antibacterial activity of the hydrolysates was studied by the disk diffusion method against Escherichia coli (BIOTECH 1634) and Staphylococcus aureus (BIOTECH 1582), and inhibition zones were not obtained. In the wild pistachio study, the inhibition diameter of the protein was found to be larger than the inhibition diameter of the hydrolysates [7]. It was reported that high amounts of Asp and Glu could reduce the antimicrobial activity of the peptides [7]. It can be thought that there is a relationship between antibacterial activity and amino acid profile. In the study conducted by Liu et al. [44], peptides were obtained by hydrolysis of walnut waste with pepsin. The purified peptide showed antibacterial activity against *E. coli* (MIC = 1.33 mg/mL) and *S. aureus* (MIC = 0.33 mg/mL). It can be said that the peptides produced by chymotrypsin hydrolysate (MIC = 0.378 mg/mL) showed similar inhibitory activity against *S. aureus* in our study. In addition, in our study, antibacterial activity was determined for pepsin hydrolysate with the agar well diffusion method (Table 4). In the study, the allergen protein Pis v 2.0101 (11S globulin), obtained by trypsin hydrolysis of defatted pistachio, showed antibacterial activity against *S. aureus* (MIC = 12 µg/mL) and *E. coli* (MIC = 50 µg/mL) [45]. The allergen protein in that study showed higher antibacterial activity than our samples. It is thought that the type of enzyme used and the hydrolysis conditions applied, which are factors affecting the bioactivity of peptides, can be effective with regard to this difference. In our study, trypsin hydrolysate showed antibacterial activity against both microorganisms by the agar well diffusion method. Walnut meal was fermented with *Lactiplantibacillus plantarum* B7 and *B. subtilis* (CICC 10002) by Hu et al. [46]. Similarly, the agar well diffusion method was used to determine the antimicrobial activity. While the produced peptides showed inhibition zones between 10.50 mm and 11.75 mm against *S. aureus*, it was stated that no inhibition zones were formed against *E. coli*. In our study, the SSF sample showed minimum inhibitory concentration of 0.776 mg/mL only against *S. aureus*. Ineffectiveness against *E.coli* can be explained by the low amount of hydrophobic amino acids. In our study, methionine, glycine, and isoleucine were detected in small amounts in the SSF sample (Table 3), while Hu et al. [46] found that peptides contain small number of amino acids such as phenylalanine and proline. Antimicrobial peptides usually have an amphiphilic structure that is the combination of cationic (lysine, histidine, and arginine) and hydrophobic amino acids [46,47]. Antimicrobial peptides can easily interact with the cytoplasmic membrane of pathogens using their charged hydrophobic structure [45]. The positive charge of antimicrobial peptides is attracted to the negatively charged membrane of microorganisms. The antimicrobial activity of a peptide is related to its hydrophobic activity since hydrophobic amino acid residues are capable of penetrating the hydrophobic core of microbial cell membranes, causing cytoplasmic leakage, membrane rupture, and cell death [46,47]. When the results are evaluated, it can be said that enzymatic hydrolysis is more efficient than solid-state fermentation in obtaining antibacterial peptides from pistachio nuts.

After gel, no growth was observed in the well for both microorganisms in groups P12, P13, P17, P18, and P21. While there was no growth of *E. coli* in the well in samples P14, P16, P23, P24, P25, P26, and P33, growth of *S. aureus* was observed. For trypsin, chymotrypsin, and savinase hydrolysate, no growth was observed in the wells in any of the samples after gel filtration.

## 4. Conclusions

In this study, pistachio waste, which is a valuable waste product, was studied. It was determined that peptides obtained by SSF, defatted pistachio, pistachio protein isolate (PPI), and enzyme hydrolysates showed antioxidant activity. These findings indicate that pistachio waste may be a good source of antioxidants. The highest ABTS radical scavenging activity was found for trypsin hydrolysate, while the maximum DPPH radical scavenging activity was obtained by hydrolysis with savinase. The highest degree of hydrolysis was obtained by using pepsin. It was observed that the type of enzyme had a significant effect on the degree of hydrolysis. Additionally, a rich amino acid content was determined in the products. The sample which had the highest TAA and EAA content was trypsin hydrolysate. The amino acid found in the uppermost amount in PPI was Asp, and it was Tyr in trypsin hydrolysate. The sample showing the highest antibacterial activity was chymotrypsin hydrolysate. Amino acid content was found to be effective on antioxidant and antimicrobial activity. When the results of enzymatic hydrolysis and SSF are compared we can conclude that peptides produced by enzymatic hydrolysis have higher antioxidant and antimicrobial activity than peptides produced by SSF. The produced peptides have the potential to be used as natural antioxidant and antimicrobial agents. Food system applications for peptides could also be possible. Bioavailability and peptide identification studies would also make great contributions to the research field. As a conclusion, we can recommend the use of enzymatic hydrolysis to obtain antimicrobial and antioxidative pistachio waste peptides. A potential use could be as an antioxidative agent to decrease lipid oxidation in meat and fish products. The use of immobilized enzymes could be feasible solution for such applications. In addition to all their benefits and advantages, the safety of bioactive peptides is also a critical issue that needs to be studied in detail. It is very important to ensure their safety, which includes the absence of toxicity and allergenicity.

## Figures and Tables

**Table 1:** Degree of hydrolysis (%) and protein concentration (mg/mL) of hydrolysates with standard deviation.

Enzyme Type	Hydrolysis Degree (%)	Protein Concentration (mg/mL)
Trypsin	16.4 ± 1.3 b	1.26 ± 0.11 b
Chymotrypsin	17.0 ± 0.9 b	1.51 ± 0.04 a
Savinase	19.1 ± 1.0 b	1.24 ± 0.05 b
Pepsin	28.2 ± 1.5 a	0.45 ± 0.02 c

Different letters on columns indicate significant differences among enzyme hydrolysate samples (*p* < 0.05).

**Table 2:** Protein concentration and antioxidant activity of samples.

Sample	Protein Concentration (mg/mL)	ABTS (µmol TE/g Defatted Pistachio, d.b.)	DPPH (µmol TE/g Defatted Pistachio, d.b.)
Defatted pistachio	19.00 ± 1.12 B	198 ± 6 BC	60.6 ± 0.8 A
Pistachio protein isolate (PPI)	31.23 ± 0.79 A	53 ± 2 D	15.7 ± 0.6 C
SSF sample with *B. subtilis*	6.21 ± 0.26 C	209 ± 7 AB	35.8 ± 1.2 B
Pepsin hydrolysate	0.45 ± 0.02 Dc	62 ± 1 D	67.3 ± 2.3 A
Trypsin hydrolysate	1.26 ± 0.11 Db	232 ± 8 A	61.9 ± 1.9 A
Chymotrypsin hydrolysate	1.51 ± 0.04 Da	177 ± 5 C	61.5 ± 2.0 A
Savinase hydrolysate	1.24 ± 0.05 Db	179 ± 5 C	70.2 ± 2.3 A

Small letters show the significant differences among enzyme hydrolysate samples; capital letters show among whole samples.

**Table 3:** Amino acid composition of pistachio protein isolate, its hydrolysates, and SSF samples (g/100 g protein).

Amino Acid	Pistachio Protein Isolate	SSF Sample with *B. subtilis*	Pepsin Hydrolysate	Trypsin Hydrolysate	Chymotrypsin Hydrolysate	Savinase Hydrolysate
Ser	1.622 ± 0.058 a	1.780 ± 0.077 a	1.045 ± 0.057 c	0.707 ± 0.029 d	1.083 ± 0.042 c	1.489 ± 0.076 b
His	3.226 ± 0.179 d	5.020 ± 0.289 a	3.652 ± 0.030 cd	3.709 ± 0.337 cd	4.466 ± 0.383 ab	4.040 ± 0.139 bc
Gly	0.091 ± 0.005 d	0.467 ± 0.018 b	0.380 ± 0.015 c	0.113 ± 0.004 d	0.439 ± 0.017 bc	0.091 ± 0.003 d
Thr	0.253 ± 0.007 b	0.341 ± 0.012 a	0.275 ± 0.008 b	0.046 ± 0.002 d	0.162 ± 0.005 c	0.334 ± 0.014 a
Arg	1.049 ± 0.041 ab	0.906 ± 0.033 bc	1.182 ± 0.033 a	0.076 ± 0.036 d	0.842 ± 0.034 c	0.825 ± 0.024 c
Ala	5.797 ± 0.337 a	6.797 ± 0.335 a	6.148 ± 0.364 a	5.990 ± 0.156 a	6.067 ± 0.134 a	5.838 ± 0.181 a
Tyr	8.440 ± 0.113 d	9.982 ± 0.332 cd	11.020 ± 0.416 bc	22.000 ± 0.749 a	8.776 ± 0.338 d	12.352 ± 0.569 b
Cys	7.097 ± 0.255 a	7.817 ± 0.340 a	7.182 ± 0.050 a	7.296 ± 0.202 a	7.302 ± 0.222 a	7.014 ± 0.223 a
Val	6.886 ± 0.353 b	8.292 ± 0.410 a	7.145 ± 0.000 ab	7.003 ± 0.140 ab	6.982 ± 0.310 b	7.185 ± 0.020 ab
Met	0.226 ± 0.008 b	0.381 ± 0.015 a	0.257 ± 0.009 b	0.153 ± 0.005 c	0.098 ± 0.003 d	0.051 ± 0.003 e
Asp	10.337 ± 0.311 a	11.951 ± 0.610 a	10.756 ± 0.002 a	10.754 ± 0.007 a	10.758 ± 0.000 a	10.758 ± 0.002 a
Glu	7.159 ± 0.191 a	8.047 ± 0.379 a	7.541 ± 0.230 a	7.457 ± 0.197 a	7.250 ± 0.209 a	7.232 ± 0.225 a
Phe	2.702 ± 0.129 ab	3.181 ± 0.211 a	2.882 ± 0.120 ab	2.654 ± 0.092 b	2.472 ± 0.049 b	2.867 ± 0.109 ab
Ile	1.199 ± 0.042 cd	1.253 ± 0.063 cd	1.181 ± 0.043 cd	1.371 ± 0.047 c	2.817 ± 0.098 a	1.639 ± 0.008 b
Leu	7.054 ± 0.197 a	8.208 ± 0.225 a	7.530 ± 0.090 a	7.336 ± 0.078 a	7.345 ± 0.180 a	7.452 ± 0.136 a
TAA	63.136 ± 2.226	74.423 ± 3.349	68.176 ± 1.467	76.665 ± 2.081	66.859 ± 2.024	69.167 ± 1.732
EAA	29.985 ± 1.028	36.658 ± 1.557	33.942 ± 0.716	44.272 ± 1.450	33.118 ± 1.366	35.920 ± 0.998
E/T (%)	47.493	49.256	49.786	57.747	49.534	51.932

Letters indicate statistical differences on rows (*p* < 0.05), showing the effect of processes on the amount of amino acids.

**Table 4:** Antibacterial activity of samples against *Escherichia coli* and *Staphylococcus aureus*.

Sample	*E. coli*	*S. aureus*
Distilled water	−	−
Pistachio protein isolate	−	+
Pepsin hydrolysate	+	+
Trypsin hydrolysate	+	+
Chymotrypsin hydrolysate	+	+
Savinase hydrolysate	+	+
Defatted pistachio	+	+
SSF sample with *B. subtilis*	−	−
Tetracycline (30 µg)	+	+

+: No microbial growth in well; −: microbial growth in well.

## Data Availability

The original contributions presented in this study are included in the article/Supplementary Materials. Further inquiries can be directed to the corresponding author.

## Supplementary Materials

The following supporting information can be downloaded at: https://www.mdpi.com/article/10.3390/foods15020392/s1. Table S1: Antioxidant activity of trypsin and chymotrypsin samples after gel filtration by DPPH and ABTS methods; Table S2: Antioxidant activity of pepsin and savinase samples after gel filtration by DPPH and ABTS methods.

## References

[1] Daliri E.B.M., Oh D.H., Lee B.H. (2017). Bioactive peptides. Foods.

[2] Sánchez A., Vázquez A. (2017). Bioactive peptides: A review. Food Qual. Saf..

[3] Mada S.B., Ugwu C.P., Abarshi M.M. (2020). Health promoting effects of food-derived bioactive peptides: A review. Int. J. Pept. Res. Ther..

[4] Singh B.P., Vij S., Hati S. (2014). Functional significance of bioactive peptides derived from soybean. Peptides.

[5] Dumandan N.G., Angelica M.R.N., Belina-Aldemita M.D., Torio M.A.O. (2014). Extraction and characterization of bioactive peptides derived from the hydrolysates of total soluble proteins of pistachio nuts (*Pistacia vera* L.). Kimika.

[6] Li P., Jia J., Fang M., Zhang L., Guo M., Xie J., Wei D. (2014). In vitro and in vivo ACE inhibitory of pistachio hydrolysates and in silico mechanism of identified peptide binding with ACE. Process Biochem..

[7] Sarabandi K., Zolqadri R., Akbarbaglu Z., Gharehbeglou P., Peighambardoust S.H., Jafari S.M. (2023). Nutritional, functional, biological and antibacterial properties of wild pistachio (*P. khinjuk*) nuts peptides. J. Food Meas. Charact..

[8] Liu C., Ren D., Li J., Fang L., Wang J., Liu J., Min W. (2018). Cytoprotective effect and purification of novel antioxidant peptides from hazelnut (*C. heterophylla* Fisch) protein hydrolysates. J. Funct. Foods.

[9] Liu C., Fang L., Min W., Liu J., Li H. (2018). Exploration of the molecular interactions between angiotensin-I-converting enzyme (ACE) and the inhibitory peptides derived from hazelnut (*Corylus heterophylla* Fisch.). Food Chem..

[10] Jamdar S.N., Rajalakshmi V., Pednekar M.D., Juan F., Yardi V., Sharma A. (2010). Influence of degree of hydrolysis on functional properties, antioxidant activity and ACE inhibitory activity of peanut protein hydrolysate. Food Chem..

[11] Ji N., Sun C., Zhao Y., Xiong L., Sun Q. (2014). Purification and identification of antioxidant peptides from peanut protein isolate hydrolysates using UHR-Q-TOF mass spectrometer. Food Chem..

[12] Liu X., Miao X., Wu D., Liu C., Fang L., Liu J., Min W. (2018). Purification and identification of ACE-inhibiting peptides from wild pine nut peptide fractions (PNPF). Eur. Food Res. Technol..

[13] Yang R., Li X., Lin S., Zhang Z., Chen F. (2017). Identification of novel peptides from 3 to 10 kDa pine nut (*Pinus koraiensis*) meal protein, with an exploration of the relationship between their antioxidant activities and secondary structure. Food Chem..

[14] Chen N., Yang H., Sun Y., Niu J., Liu S. (2012). Purification and identification of antioxidant peptides from walnut (*Juglans regia* L.) protein hydrolysates. Peptides.

[15] Feng L., Peng F., Wang X., Li M., Lei H., Xu H. (2019). Identification and characterization of antioxidative peptides derived from simulated in vitro gastrointestinal digestion of walnut meal proteins. Food Res. Int..

[16] Derbyshire E., Higgs J., Feeney M.J., Carughi A. (2023). Believe it or ‘nut’: Why it is time to set the record straight on nut protein quality: Pistachio (*Pistacia vera* L.) focus. Nutrients.

[17] Salinas M.V., Guardianelli L.M., Sciammaro L.P., Picariello G., Mamone G., Puppo M.C. (2021). Nutritional ingredient by-product of the pistachio oil industry: Physicochemical characterization. J. Food Sci. Technol..

[18] Bulló M., Juanola-Falgarona M., Hernández-Alonso P., Salas-Salvadó J. (2015). Nutrition attributes and health effects of pistachio nuts. Br. J. Nutr..

[19] Mirzapour M., Rezaei K., Sentandreu M.A., Moosavi-Movahedi A.A. (2016). In vitro antioxidant activities of hydrolysates obtained from Iranian wild almond (*Amygdalus scoparia*) protein by several enzymes. Int. J. Food Sci. Technol..

[20] Sarmadi B.H., Ismail A. (2010). Antioxidative peptides from food proteins: A review. Peptides.

[21] Toldrá F., Reig M., Aristoy M.C., Mora L. (2018). Generation of bioactive peptides during food processing. Food Chem..

[22] Ferreira C.D., Ziegler V., da Silva Lindemann I., Hoffmann J.F., Vanier N.L., de Oliveira M. (2018). Quality of black beans as a function of long-term storage and moldy development: Chemical and functional properties of flour and isolated protein. Food Chem..

[23] Nielsen P.M., Petersen D., Dambmann C. (2001). Improved method for determining food protein degree of hydrolysis. J. Food Sci..

[24] Spellman D., McEvoy E., O’cuinn G., FitzGerald R.J. (2003). Proteinase and exopeptidase hydrolysis of whey protein: Comparison of the TNBS, OPA and pH stat methods for quantification of degree of hydrolysis. Int. Dairy J..

[25] Silvestre M.P.C., Morais H.A., Silva V.D.M., Silva M.R. (2013). Degree of hydrolysis and peptide profile of whey proteins using pancreatin. Nutr. Rev. Soc. Bras. Aliment. Nutr..

[26] Bradford M.M. (1976). A rapid and sensitive method for the quantitation of microgram quantities of protein utilizing the principle of protein-dye binding. Anal. Biochem..

[27] Wu W., Zhao S., Chen C., Ge F., Liu D., He X. (2014). Optimization of production conditions for antioxidant peptides from walnut protein meal using solid-state fermentation. Food Sci. Biotechnol..

[28] Jiang X., Cui Z., Wang L., Xu H., Zhang Y. (2020). Production of bioactive peptides from corn gluten meal by solid-state fermentation with Bacillus subtilis MTCC5480 and evaluation of its antioxidant capacity in vivo. LWT.

[29] Brand-Williams W., Cuvelier M.E., Berset C.L.W.T. (1995). Use of a free radical method to evaluate antioxidant activity. LWT-Food Sci. Technol..

[30] Gu M.U., Chen H.P., Zhao M.M., Wang X.I., Yang B., Ren J.Y., Su G.W. (2015). Identification of antioxidant peptides released from defatted walnut (*Juglans Sigillata Dode*) meal proteins with pancreatin. LWT-Food Sci. Technol..

[31] Balouiri M., Sadiki M., Ibnsouda S.K. (2016). Methods for in vitro evaluating antimicrobial activity: A review. J. Pharm. Anal..

[32] Clinical and Laboratory Standards Institute (2012). Performance Standards for Antimicrobial Disk Susceptibility Tests; Approved Standard-Eleventh Edition.

[33] Clinical and Laboratory Standards Institute (2012). Methods for Dilution Antimicrobial Susceptibility Tests for Bacteria that Grow Aerobically; Approved Standard—Ninth Edition.

[34] Jajić I., Krstović S., Glamočić D., Jakšić S., Abramović B. (2013). Validation of an HPLC method for the determination of amino acids in feed. J. Serbian Chem. Soc..

[35] KabeloVá I., DVořáKoVá M., ČížKoVá H., DoStáleK P., MelzoCH K. (2009). Determination of free amino acids in cheeses from the Czech market. Czech J. Food Sci.

[36] Ghribi A.M., Sila A., Przybylski R., Nedjar-Arroume N., Makhlouf I., Blecker C., Besbes S. (2015). Purification and identification of novel antioxidant peptides from enzymatic hydrolysate of chickpea (*Cicer arietinum* L.) protein concentrate. J. Funct. Foods.

[37] Sealey-Voyksner J., Zweigenbaum J., Voyksner R. (2016). Discovery of highly conserved unique peanut and tree nut peptides by LC–MS/MS for multi-allergen detection. Food Chem..

[38] Zhang Y., Wu Z., Li K., Li X., Yang A., Tong P., Chen H. (2019). Allergenicity assessment on thermally processed peanut influenced by extraction and assessment methods. Food Chem..

[39] D’Evoli L., Lucarini M., Gabrielli P., Aguzzi A., Lombardi-Boccia G. (2015). Nutritional value of Italian pistachios from Bronte (*Pistacia vera*, L.), their nutrients, bioactive compounds and antioxidant activity. Food Nutr. Sci..

[40] Gentile C., Tesoriere L., Butera D., Fazzari M., Monastero M., Allegra M., Livrea M.A. (2007). Antioxidant activity of Sicilian pistachio (*Pistacia vera* L. var. Bronte) nut extract and its bioactive components. J. Agric. Food Chem..

[41] Hu F., Ci A.T., Wang H., Zhang Y.Y., Zhang J.G., Thakur K., Wei Z.J. (2018). Identification and hydrolysis kinetic of a novel antioxidant peptide from pecan meal using Alcalase. Food Chem..

[42] Zheng L., Zhao Y., Xiao C., Sun-Waterhouse D., Zhao M., Su G. (2015). Mechanism of the discrepancy in the enzymatic hydrolysis efficiency between defatted peanut flour and peanut protein isolate by Flavorzyme. Food Chem..

[43] World Health Organization, United Nations University (2007). Protein and Amino Acid Requirements in Human Nutrition.

[44] Liu D., Liu M., Meng D., Mu Y., Wang T., Lv Z. (2022). Harsh sensitivity and mechanism exploration of an antibacterial peptide extracted from walnut oil residue derived from agro-industrial waste. J. Agric. Food Chem..

[45] Taghizadeh S.F., Rezaee R., Mehmandoust M., Badibostan H., Karimi G. (2020). Assessment of in vitro bioactivities of Pis v 1 (2S albumin) and Pis v 2.0101 (11S globulin) proteins derived from pistachio (*Pistacia vera* L.). J. Food Meas. Charact..

[46] Hu Y., Ling Y., Qin Z., Huang J., Jian L., Ren D.F. (2024). Isolation, identification, and synergistic mechanism of a novel antimicrobial peptide and phenolic compound from fermented walnut meal and their application in *Rosa roxbughii* Tratt spoilage fungus. Food Chem..

[47] Nguyen L.T., Haney E.F., Vogel H.J. (2011). The expanding scope of antimicrobial peptide structures and their modes of action. Trends Biotechnol..

