# Supplementary material for: Antioxidant and Antibacterial Activity, and the Amino Acid Profile of Pistachio (Pistacia vera L.) Waste Peptides Produced by Enzymatic Hydrolysis and Solid-State Fermentation
Source: Foods. 2026 Jan 22;15(2):392. doi: 10.3390/foods15020392 (PMC12841511; doi:10.3390/foods15020392)
Supplement: Supplementary file 1 [file foods-15-00392-s001.zip › foods-3965270-supplementary.pdf]

Supplementary Material

Table S1. Antioxidant activity of trypsin and chymotrypsin samples after gel filtration by DPPH and ABTS methods

Sample	Protein concentration (mg/ml)	DPPH radical scavenging activity (mg Trolox eqv/ml)	ABTS radical scavenging activity (mg Trolox eqv/ml)	Sample	Protein concentration (mg/ml)	DPPH radical scavenging activity (mg Trolox eqv/ml)	ABTS radical scavenging activity (mg Trolox eqv/ml)
T6	0.3123 ± 0.0262a	0.0511 ± 0.0009ab	0.0757 ± 0.0049b	C7	0.0367 ± 0.0023defgh	0.0244 ± 0.0005ghi	0.0127 ± 0.0009g
T7	0.3123 ± 0.0169a	0.0518 ± 0.0003a	0.1166 ± 0.0099a	C8	0.3096 ± 0.0156a	0.0562 ± 0.0002a	0.0793 ± 0.0014a
T8	0.2756 ± 0.0112a	0.0498 ± 0.0002bc	0.0765 ± 0.0031b	C9	0.2240 ± 0.0130b	0.0558 ± 0.0002a	0.0297 ± 0.0025d
T9	0.1792 ± 0.0134b	0.0491 ± 0.0001c	0.0501 ± 0.0051c	C10	0.2220 ± 0.0029b	0.0552 ± 0.0005a	0.0699 ± 0.0019b
T10	0.0872 ± 0.0037c	0.0238 ± 0.0005ef	0.0443 ± 0.0037c	C11	0.0815 ± 0.0000c	0.0283 ± 0.0005cd	0.0492 ± 0.0025c
T11	0.0741 ± 0.0000cd	0.0222 ± 0.0001fg	0.0259 ± 0.0030d	C12	0.0562 ± 0.0050cdef	0.0309 ± 0.0004b	0.0267 ± 0.0025e
T12	0.0525 ± 0.0006cdef	0.0207 ± 0.0004gh	0.0207 ± 0.0022de	C13	0.0697 ± 0.0087cd	0.0280 ± 0.0008d	0.0296 ± 0.0031d
T13	0.0469 ± 0.0029cdefg	0.0209 ± 0.0001gh	0.0128 ± 0.0005def	C14	0.0499 ± 0.0054cdefg	0.0304 ± 0.0001b	0.0202 ± 0.0011f
T14	0.0126 ± 0.0006efg	0.0179 ± 0.0009ij	0.0146 ± 0.0016def	C15	0.0700 ± 0.0083cd	0.0263 ± 0.0020ef	0.0303 ± 0.0018d
T15	0.0285 ± 0.0011defg	0.0222 ± 0.0005fg	0.0191 ± 0.0012def	C16	0.0754 ± 0.0029c	0.0274 ± 0.0008de	0.0270 ± 0.0014e
T16	0.0416 ± 0.0025defg	0.0195 ± 0.0008hi	0.0076 ± 0.0006def	C17	0.0672 ± 0.0052cd	0.0299 ± 0.0000b	0.0146 ± 0.0005g
T17	0.0407 ± 0.0022defg	0.0235 ± 0.0004ef	0.0102 ± 0.0000def	C18	0.0486 ± 0.0043cdefg	0.0296 ± 0.0003bc	0.0093 ± 0.0007h
T18	0.0591 ± 0.0036cde	0.0248 ± 0.0015e	0.0043 ± 0.0004ef	C19	0.0192 ± 0.0018gh	0.0258 ± 0.0027fg	0.0072 ± 0.0006hi
T20	0.0350 ± 0.0011defg	0.0186 ± 0.0011ij	0.0040 ± 0.0002ef	C20	0.0204 ± 0.0123fgh	0.0221 ± 0.0022jk	0.0017 ± 0.0002j
T21	0.0224 ± 0.0012efg	0.0182 ± 0.0015ij	0.0057 ± 0.0004ef	C21	0.0285 ± 0.0023efgh	0.0211 ± 0.0009k	0.0064 ± 0.0003i
T22	0.0299 ± 0.0012defg	0.0182 ± 0.0017ij	0.0003 ± 0.0000ef	C22	0.0261 ± 0.0011efgh	0.0264 ± 0.0015ef	0.0030 ± 0.0002j
T23	0.0274 ± 0.0006efg	0.0163 ± 0.0015j	0.0087 ± 0.0005def	C23	0.0094 ± 0.0006h	0.0233 ± 0.0016ij	0.0000 ± 0.0000j
T24	0.0106 ± 0.0011fg	0.0282 ± 0.0001d	0.0124 ± 0.0011def	C24	0.0103 ± 0.0005h	0.0237 ± 0.0018hij	0.0075 ± 0.0006hi
T25	0.0135 ± 0.0010efg	0.0195 ± 0.0013hi	0.0153 ± 0.0013def	C25	0.0094 ± 0.0006h	0.0237 ± 0.0004hij	0.0031 ± 0.0003j
T27	0.0106 ± 0.0010fg	0.0230 ± 0.0012ef	0.0048 ± 0.0004ef	C35	0.0587 ± 0.0050cde	0.0230 ± 0.0003ij	0.0000 ± 0.0000j
				C36	0.0084 ± 0.0004h	0.0230 ± 0.0014ij	0.0000 ± 0.0000j
				C37	0.0255 ± 0.0074efgh	0.0275 ± 0.0013de	0.0000 ± 0.0000j
				C38	0.0262 ± 0.0012efgh	0.0240 ± 0.0012hi	0.0000 ± 0.0000j
				C39	0.0298 ± 0.0029efgh	0.0250 ± 0.0002fgh	0.0000 ± 0.0000j

Different letters on columns indicate significant differences among samples ($p < 0.05$)

Table S2. Antioxidant activity of pepsin and savinase samples after gel filtration by DPPH and ABTS methods

Sample	Protein concentration (mg/ml)	DPPH radical scavenging activity (mg Trolox eqv/ml)	ABTS radical scavenging activity (mg Trolox eqv/ml)	Sample	Protein concentration (mg/ml)	DPPH radical scavenging activity (mg Trolox eqv/ml)	ABTS radical scavenging activity (mg Trolox eqv/ml)
P8	0.0457 ± 0.0046c	0.0245 ± 0.0018fghi	0.0000 ± 0.0000f	S7	0.0167 ± 0.0014ghi	0.0253 ± 0.0009cdef	0.0042 ± 0.0003def
P9	0.1141 ± 0.0078b	0.0249 ± 0.0023fghi	0.0121 ± 0.0013b	S8	0.3302 ± 0.0392a	0.0573 ± 0.0003a	0.0797 ± 0.0072a
P10	0.1988 ± 0.0122a	0.0273 ± 0.0005f	0.0180 ± 0.0013a	S9	0.2183 ± 0.0202b	0.0560 ± 0.0002a	0.0700 ± 0.0049a
P11	0.0338 ± 0.0016c	0.0304 ± 0.0005e	0.0197 ± 0.0017a	S10	0.1483 ± 0.0106c	0.0538 ± 0.0026a	0.0768 ± 0.0015a
P12	0.0331 ± 0.0000c	0.0256 ± 0.0005fgh	0.0113 ± 0.0011b	S11	0.1466 ± 0.0092c	0.0552 ± 0.0001a	0.0522 ± 0.0032b
P13	0.0196 ± 0.0010c	0.0266 ± 0.0019f	0.0076 ± 0.0006c	S12	0.0733 ± 0.0046d	0.0197 ± 0.0013h	0.0454 ± 0.0023b
P14	0.0322 ± 0.0030c	0.0303 ± 0.0004e	0.0059 ± 0.0004cde	S13	0.0347 ± 0.0032efghi	0.0311 ± 0.0009b	0.0127 ± 0.0009cdef
P15	0.0253 ± 0.0012c	0.0427 ± 0.0005b	0.0060 ± 0.0003cde	S14	0.0697 ± 0.0040de	0.0226 ± 0.0014efgh	0.0203 ± 0.0002c
P16	0.0377 ± 0.0024c	0.0472 ± 0.0013a	0.0184 ± 0.0015a	S15	0.0456 ± 0.0031defgh	0.0292 ± 0.0005bc	0.0145 ± 0.0013cdef
P17	0.0415 ± 0.0030c	0.0390 ± 0.0007c	0.0178 ± 0.0020a	S16	0.0499 ± 0.0036defgh	0.0260 ± 0.0012cdef	0.0198 ± 0.0008c
P18	0.0163 ± 0.0012c	0.0339 ± 0.0013d	0.0074 ± 0.0006cd	S17	0.0524 ± 0.0048defg	0.0257 ± 0.0018cdef	0.0175 ± 0.0017cde
P21	0.0098 ± 0.0000c	0.0218 ± 0.0022ij	0.0014 ± 0.0001f	S18	0.0530 ± 0.0042defg	0.0258 ± 0.0001cdef	0.0185 ± 0.0005cd
P22	0.0102 ± 0.0006c	0.0227 ± 0.0017hij	0.0047 ± 0.0005e	S19	0.0624 ± 0.0040def	0.0273 ± 0.0006cd	0.0175 ± 0.0018cde
P23	0.0122 ± 0.0011c	0.0235 ± 0.0005ghij	0.0064 ± 0.0006cde	S20	0.0415 ± 0.0034defgh	0.0228 ± 0.0018efgh	0.0141 ± 0.0002cdef
P24	0.0261 ± 0.0000c	0.0263 ± 0.0019fg	0.0049 ± 0.0005de	S21	0.0212 ± 0.0013ghi	0.0263 ± 0.0006cde	0.0046 ± 0.0000def
P25	0.0126 ± 0.0006c	0.0210 ± 0.0018j	0.0052 ± 0.0006cde	S22	0.0245 ± 0.0011fghi	0.0232 ± 0.0018efgh	0.0028 ± 0.0002ef
P26	0.0285 ± 0.0011c	0.0227 ± 0.0016hij	0.0000 ± 0.0000f	S23	0.0388 ± 0.0071defghi	0.0237 ± 0.0017defg	0.0099 ± 0.0006cdef
P33	0.0114 ± 0.0000c	0.0234 ± 0.0014ghij	0.0000 ± 0.0000f	S29	0.0620 ± 0.0022def	0.0241 ± 0.0022defg	0.0015 ± 0.0001f
P34	0.0375 ± 0.0011c	0.0213 ± 0.0012j	0.0000 ± 0.0000f	S30	0.0138 ± 0.0000hi	0.0273 ± 0.0006cd	0.0027 ± 0.0002ef

Different letters on columns indicate significant differences among samples (p < 0.05)
